# A Multi-Component Nutraceutical Formulation for the Management of Vascular and Inflammatory Alterations Characteristic of the Oedema Disorders

**DOI:** 10.3390/nu18030523

**Published:** 2026-02-04

**Authors:** Maria Maisto, Adua Marzocchi, Vincenzo Piccolo, Roberto Ciampaglia, Marlo De Vivo, Gian Carlo Tenore

**Affiliations:** NutraPharmaLab, Department of Pharmacy, University of Naples Federico II, Via Domenico Montesano 59, 80131 Naples, Italy; maria.maisto@unina.it (M.M.); adua.marzocchi@unina.it (A.M.); roberto.ciampaglia@unina.it (R.C.); marlodevivo604@gmail.com (M.D.V.); giancarlo.tenore@unina.it (G.C.T.)

**Keywords:** macrophage polarisation, alternative remedies, vascular functionality, paw oedema, oedema-associated inflammation

## Abstract

**Background**: Oedema is a multifactorial condition arising from the interplay between increased microvascular permeability, impaired lymphatic clearance, and sustained inflammation. Conventional treatments often fail, highlighting alternative therapies. This study explores a novel nutraceutical formulation (NF) based on the combination of different natural extracts, i.e., *Melilotus officinalis* L., *Olea europaea* L., *Morinda citrifolia* L., *Quercus robur* L., and bromelain, aimed at reducing inflammation, a key contributor to oedema pathophysiology. In vitro assays further demonstrated that NF exhibits a marked antioxidant capacity and effectively inhibits key enzymes of the arachidonic acid cascade, supporting its ability to counteract oxidative stress and inflammatory signalling involved in oedema pathophysiology. **Methods**: The antioxidant and anti-inflammatory properties of NF were assessed in vitro using radical scavenging assays and enzyme inhibition tests targeting key components of the arachidonic acid cascade. The immunomodulatory effects of NF were investigated in RAW264.7 macrophages by flow cytometry and RT-qPCR to evaluate macrophage polarisation and cytokine expression. The anti-oedematous and vascular effects were further examined in vivo using acetic acid–induced inflammation and carrageenan-induced paw oedema models in thirty male Sprague–Dawley rats (Charles River, Calco, Italy). **Results**: The study demonstrated that NF significantly modulates macrophage polarisation, reducing the proportion of pro-inflammatory M1 macrophages (F4/80^+^CD11b^+^) by 3.23 times compared to control (*p* < 0.01). A quantitative PCR analysis further confirmed a decrease in pro-inflammatory cytokines (TNF-α, IL-6, and IL-1β) by 51.3% (95% CI 48.0–58.7, *p* < 0.001), 64.1% (95% CI 57.0–71.2, *p* < 0.001), and 53.7% (95% CI 51.7–55.7, *p* < 0.001), respectively compared to the control, while anti-inflammatory markers (Arg-1, CD206) increased significantly, suggesting a shift towards an M2 anti-inflammatory state. The NF ability to contrast the pathological alteration characteristic of this disease was further tested in the rat oedema model of thirty male Sprague-Dawley rats. The NF treatment reduced LTB4 and plasma protein levels compared to the control group. In addition, NF could decrease the paw thickness in the rat-based carrageenan-induced oedema model (Charles River, Calco, Italy; *n* = 30) by 22.5% compared to the control (95% CI 11.0–34.0, *p* < 0.05). **Conclusions**: These results suggest that NF may provide a multi-target approach to support the management of some physiopathological changes in complex oedema-related conditions by both modulating inflammation and restoring vascular functionality.

## 1. Introduction

Oedema is defined as a visible swelling caused by an abnormal expansion of interstitial fluid volume within tissues or organs [1]. Under physiological conditions, tissue fluid homeostasis is regulated by the dynamic balance between microvascular filtration, the integrity of the endothelial barrier, and fluid return to the circulation through lymphatic drainage [2]. Accordingly, oedema develops whenever higher filtration persistently exceeds lymphatic clearance, with mechanisms that include increased capillary hydrostatic pressure, reduced plasma oncotic pressure, altered endothelial permeability, and impaired lymphatic outflow [3]. A pivotal driver of this pathological state is the activation of a localised inflammatory response. Specifically, the accumulation of extravasated interstitial fluid promotes the recruitment and retention of cytokines, chemokines, and leukocyte subsets, fostering a sustained pro-inflammatory state that further alters microvascular function [4]. From a mechanistic standpoint, inflammation-induced extravasation is closely linked to the structural alteration of the microvascular barrier, encompassing the degradation of the endothelial glycocalyx, disruption of tight and adherens junctional complexes, and remodelling of the basement membrane. These alterations markedly enhance paracellular permeability, facilitating the leakage of water, plasma proteins, and inflammatory mediators into tissue [5].

Clinically, oedema can be categorised according to its anatomical distribution, chronicity, and underlying pathophysiology [1]. It is traditionally classified as localised oedema, affecting a specific anatomical region, or generalised oedema (anasarca), which reflects systemic fluid overload [6]. Several distinct forms of oedema are described. Venous oedema is the most common and results from elevated venous pressure, such as in chronic venous insufficiency, which increases capillary filtration and enhances the fluid accumulation at the tissue level [1,3]. Inflammatory oedema, instead, arises when pro-inflammatory mediators alter endothelial junctions, leading to marked increases in vascular permeability and rapid extravasation of protein-rich fluid [7,8]. Lymphatic oedema (lymphoedema) develops when lymphatic transport capacity is reduced, preventing adequate clearance of interstitial fluid and macromolecules and progressively leading to tissue fibrosis [9]. Finally, hypoproteinaemic oedema occurs when low plasma protein levels, particularly hypoalbuminaemia, decrease oncotic pressure and impair vascular fluid retention [1,10]. In many clinical contexts, the pathophysiology of oedema does not derive from a single causal mechanism. Although oedema is conventionally classified as venous, inflammatory, lymphatic, or hypoproteinaemic, these processes often occur in combination within the same patient. This pattern is particularly evident in the lower limbs, where venous hypertension, inflammation-mediated increases in endothelial permeability, and lymphatic insufficiency frequently coexist and produce composite phenotypes in which each alteration amplifies the others. Independent of the predominant clinical subtype, a unifying feature of oedema is the convergence of vascular and immunological abnormalities that elevate endothelial permeability, permit extravasation of plasma proteins, and favour leukocyte recruitment, ultimately sustaining chronic interstitial fluid accumulation [11]. Despite the multifactorial nature of oedema, its therapeutic management remains challenging, as effective control requires simultaneous modulation of endothelial hyperpermeability, inflammatory signalling, and impaired fluid clearance. Most available therapies, however, target only one pathogenic axis, resulting in incomplete correction of the underlying microvascular dysfunction. Current management is therefore primarily aetiology-driven, combining treatment of systemic contributors (such as cardiac, renal, or hepatic disease) with local strategies to enhance venous and lymphatic return and reduce capillary filtration [12]. Compression therapy is broadly effective across many lower-extremity oedema phenotypes, whereas diuretics are reserved for oedema secondary to systemic fluid overload rather than nonsystemic presentations. Specifically, in lymphoedema, conservative care centres on complete decongestive therapy, including manual lymphatic drainage, multilayer compression, and skin care, which together support lymphatic transport and mitigate chronic tissue changes [13]. Recently, great attention has been paid to the identification of natural remedies for the alternative treatment of such chronic conditions [14,15,16,17,18,19,20]. Traditionally, various natural remedies have been widely used to treat altered vascular fluid balance and related inflammation. Given the multifactorial nature of this condition, the most extensively studied natural remedies are multi-targeted and address different mechanisms of action. These remedies work either through two different alternative mechanisms or by reducing vascular permeability or mitigating the local inflammatory process.

Specifically, in available literature is reported that coumarin (5,6-benzo-alpha pyrone), a natural compound extracted from *Melilotus officinalis* L., has shown valuable activity in enhancing the function of vessels and capillaries, reducing in this way protein accumulation at the lymphatic level [21]. Interestingly, a recent clinical trial has investigated the synergic activity of *Melilotus officinalis* L. extract (titrated in coumarin), in combination with rutin and bromelain, on 52 enrolled subjects affected by primary or secondary lymphoedema [22]. After two months of treatment, the authors reported a valuable reduction in the main symptoms related to lymphoedema, including a reduction in limb circumference of 4.2 cm, an average decrease in superficial thickness by 29% in comparison to T0, suggesting potential utility in oedema conditions where lymphatic dysfunction contributes [22]. Additionally, coumarin-containing extracts of *Melilotus officinalis* L. have been evaluated for oedema related to phlebo-lymphatic stasis, supporting the rationale for targeting microcirculatory function in oedematous states [23]. The relevance of natural compounds in the treatment of lymphoedema diseases was corroborated by another piece of clinical evidence, which described the protective activity of tannin-based supplementation on the main lymphoedema symptoms [24]. Their results underline that a significant reduction in leg volume by −18.9% in comparison to T0 after only 8 weeks of treatment, and simultaneously a valuable decrease in protein concentration in interstitial fluids by −36.9%, were described in treated patients [24]. An alternative approach to treat symptoms associated with the accumulation of liquid at the interstitial level is to modulate the associated inflammatory process. Numerous reports of natural substances modulate the inflammatory process at various levels. From traditional medicine, the leaf extract from *Morinda citrifolia* L. has shown valuable anti-inflammatory activity. It was reported that *Morinda citrifolia* L. extract can downregulate pro-inflammatory cytokines, including interleukin-6 (IL-6), interleukinaturaln-1β (IL-1β), and tumour necrosis factor-alpha (TNF-α), which are frequently elevated in oedematous tissues. Several pieces of evidence reported that *Morinda citrifolia* L. extract also exhibits valuable anti-fibrotic properties, potentially through the inhibition of profibrotic cytokines such as transforming growth factor-beta 1 (TGF-β1) [25]. This action could lead to a reduction in collagen deposition, a critical step in preventing tissue hardening and fibrosis.

In the same manner, hydroxytyrosol, the main polyphenolic component of olive leaves (*Olea europea* L.), has been largely investigated for its key role in preventing vascular inflammation and leakage by inhibiting the leukotriene B4 (LTB4) activity. LTB4, a potent lipid mediator derived from arachidonic acid, is known for its pro-inflammatory potential, including the recruitment and activation of leukocytes to sites of tissue injury or immune activation. In lymphoedema, elevated levels of LTB4 contribute to chronic inflammation within lymphatic tissues, promoting fibrosis and exacerbating lymphatic vessel dysfunction. Finally, bromelain derived from *Ananas comosus* L. is widely described as a proteolytic complex with anti-inflammatory and anti-oedematous activities across experimental and clinical literature, making it a complementary component in oedema-oriented formulations [26,27]. Thus, given that oedema arises from concurrent vascular dysfunction and inflammatory activation, we hypothesised that a multicomponent nutraceutical capable of targeting multiple pathogenic pathways could offer a more effective therapeutic strategy. On this basis, the present study aims to develop an innovative multicomponent formulation combining different bioactive compounds specifically selected to modulate the key physiopathological alterations underlying oedema, including increased vascular permeability and sustained inflammation. Specifically, the formulations will incorporate aqueous extracts of *Morinda citrifolia* L., *Melilotus officinalis* L., *Olea europaea* L., and *Quercus robur* L. These natural extracts will be combined with bromelain, an enzymatic complex derived from pineapple (*Ananas comosus* L.), known for its well-documented anti-oedematous and anti-inflammatory properties.

## 2. Materials and Methods

### 2.1. Reagents

All the extracts used for the NF preparation were supplied and certified by Anvest Health s.p.a. (Milan, Italy). Specifically, all the plant materials used in the NF, registered as Edemix^®^ (Istanbul, Turkey), are aqueous extracts differently characterised: *Melilotus officinalis* L. was titrated in 15% of coumarins, *Olea europaea* L. titrated at 10% of hydroxytyrosol, *Morinda citrifolia* L. extract titrated at 1% of rubiadin, bromelain 5000 Gelatin Dissolving Units (GDU)/g, and *Quercus robur* L. bark aqueous extract titrated at 10% of tannins. Specifically, all these components were combined to prepare a potential oral nutraceutical formulation containing: 10.4 mg, 10.4 mg, 15.6 mg, 32.2 mg, and 10.4 mg of *Morinda citrifolia* L., *Quercus robur* L., *Melilotus officinalis* L., Bromelain, and *Olea Europea* L., respectively.

### 2.2. Antioxidant Activity

#### 2.2.1. DPPH Assay

The antioxidant capacity of the samples was assessed using the DPPH (2,2-diphenyl-1-picrylhydrazyl, CAS: 1898-66-4) radical scavenging assay, as previously outlined [28]. A total of 200 µL of hydroalcoholic extract was added to 1000 µL of a 0.05 mM DPPH methanolic solution, and the mixture was incubated in the dark for 10 min. After the reaction time, the reduction in absorbance was recorded at 517 nm using a V-730 UV–Visible/NIR spectrophotometer with the Spectra Manager™ v2.5 Suite software (Jasco Inc., Easton, MD, USA). The absorbance of the DPPH solution without extract served as the blank solution. The percentage of inhibition was calculated using the equation:

$$\%\;\mathrm{inhibition}\;=\;\frac{1-Af}{Ac}\times 100$$

where *Af* is the absorbance after 10 min, and *Ac* is the absorbance of the control at time zero. 6-hydroxy-2,5,7,8-tetramethylchroman-2-carboxylic acid (Trolox, CAS: 53188-07-1) was used as the reference antioxidant, and a calibration curve was generated using eight concentrations ranging from 5 to 250 µM. Each concentration was tested in triplicate using dilution factors of 1:2 and 1:5. The extracts were analysed in triplicate in a concentration range of 0.2 to 2.75 mg/mL, and the results were expressed as IC_50_ values.

#### 2.2.2. ABTS Assay

The ABTS assay was employed to assess the ability of the extracts to quench ABTS (2,2′-azino-bis-(3-ethylbenzothiazoline-6-sulfonic acid), CAS: 30931-67-0) radical, based on the method reported by Iannuzzo et al. (2022) [28]. The ABTS working solution was prepared by mixing 2.5 mL of 7.0 mM ABTS with 0.044 mL of 140 mM potassium persulfate (CAS: 7727-21-1). This solution was incubated in the dark at 5 °C for 7 h. Subsequently, the solution was diluted with ethanol until an absorbance of 0.70 ± 0.05 at 754 nm was achieved (Jasco Inc., Easton, MD, USA). For the assay, 0.10 mL of the hydroalcoholic extract was mixed with 1 mL of the ABTS ethanolic solution and incubated in the dark for 2.5 min. The reduction in absorbance was then recorded at 517 nm. The ethanol-diluted ABTS solution without a sample was used as the blank. Inhibition percentage was determined using the formula:

$$\%\;\mathrm{inhibition}\;=\;\frac{1-Af}{Ac}\times 100$$

where *Af* is the absorbance after 10 min, and *Ac* is the absorbance of the control at time zero. Trolox served as the reference antioxidant. The calibration curve included eight concentrations ranging from 5 to 200 µM, with dilution ratios of 1:2 and 1:5, and three replicates for each. All the sample measurements were performed in triplicate in a concentration range of 0.2 to 7.5 mg/mL, and the results were expressed as IC_50_ values, representing the concentration of extract needed to inhibit 50% of the initial ABTS radical activity.

### 2.3. Anti-Inflammatory Activity

#### 2.3.1. Lipoxygenase Inhibitory Activity Assay

The lipoxygenase inhibitory activity assay was performed according to the method reported by Iannuzzo et al., with slight modifications [28]. Briefly, 125 µL of an NF solution at several concentrations was added to 125 µL of soybean lipoxidase enzyme solution (final concentration of 1250 U/mL; CAS: 9029-60-1). This mixture was incubated at 25 °C for 5 min. Then 500 µL of linoleic acid solution (358 µM; CAS: 60-33-3) was added, and the mixture was incubated for 10 min at 25 °C. A 0.2 M borate buffer solution (pH 9) was used to dissolve all the components of the assay, and 750 µL of buffer was also used to dilute the final mixture. After mixing thoroughly, the absorbance was measured at 234 nm. The percentage (%) inhibition was calculated according to the following equation:

$$\%\;\mathrm{inhibition}\;=\;\frac{Activity\;of\;LOX\;-\;Activity\;of\;LOX\;with\;sample}{Activity\;of\;LOX}\times 100$$

The results were expressed as IC_50_, which is the concentration of inhibitor needed to inhibit 50% of the enzyme. Zileuton (CAS: 111406-87-2) was used as the reference anti-inflammatory compound.

#### 2.3.2. Cyclooxygenase 1 (COX-1) and Cyclooxygenase 2 (COX-2) Inhibitory Activity Assay

The cyclooxygenase 1 (COX-1) and cyclooxygenase 2 (COX-2) inhibitory activity assays were performed using a Cayman Chemical COX Colorimetric Inhibitor Screening Assay Kit (Cayman Chemical, Ann Arbor, MI, USA, code 760151) [28]. The method assesses the peroxidase activity of COXs by a colorimetric detection of the oxidised N,N,N’,N’-tetramethyl-p-phenylenediamine (TMPD) at 590 nm. Samples were divided into a positive control (100% of COX activity), containing 150 µL of 0.1 M Tris–HCl buffer (pH 8.0), 10 µL of heme, and 10 µL of enzyme, and inhibitory samples, containing 150 µL of buffer, 10 µL of heme, 10 µL of enzyme, and 10 µL of sample solution at different concentrations. Samples were incubated at 25 °C for 5 min, and then 20 µL of arachidonic acid (AA) solution and 20 µL of a colorimetric substrate solution (TMPD) were added. After 2 min of incubation at 25 °C, the absorbance at 590 nm was read. The COX-1 and COX-2 inhibitory activities were calculated according to the following equation:

$$\%\;\mathrm{inhibition}\;=\;\frac{Activity\;of\;COX\;-\;Activity\;of\;COX\;with\;sample}{Activity\;of\;COX}\times 100$$

The results were expressed as IC_50_, which is the concentration of inhibitor needed to inhibit 50% of the enzyme. Naproxen (CAS: 22204-53-1) was used as the reference anti-inflammatory compound.

### 2.4. Cell Culture and M1 Polarisation

RAW264.7 murine macrophages (American Type Culture Collection (ATCC)) were cultured in Dulbecco’s Modified Eagle Medium (DMEM; Gibco, Grand Island, NY, USA) supplemented with 10% foetal bovine serum (FBS; Gibco, USA) and maintained at 37 °C in a humidified incubator with 5% CO_2_ (HF-90, Lishen Co., Shanghai, China). Three groups were established for the experimental setup. In the M0 group, RAW264.7 cells were maintained under standard culture conditions without stimulation. In the M1 group, the cells were treated with 500 ng/mL lipopolysaccharide (LPS; Sigma-Aldrich, St. Louis, MO, USA; CAS: 93572-42-0) and 20 ng/mL interferon-gamma (IFN-γ; Sigma-Aldrich, St. Louis, MO, USA) for 12 h to induce classical pro-inflammatory activation. Morphological changes were assessed by light microscopy (IX53, Olympus, Tokyo, Japan), and only the cells in optimal condition were selected for subsequent steps. In the NF group, M1-polarised macrophages were further treated with 40 μg/mL (identified as non-cytotoxic, maintaining over 90% cell viability compared to untreated controls in the MTT-test) of the nutraceutical formulation (NF) and incubated for an additional 24 h. After the incubation period, cells from all groups were collected for downstream analyses [29].

#### 2.4.1. M1 Macrophage Detection by Flow Cytometry

Briefly, following the treatment, the cells were collected by centrifugation at 350× *g* for 5 min at 4 °C. The cell pellets from each experimental group were carefully resuspended and washed twice with cold phosphate-buffered saline (PBS) to remove residual media and serum proteins. After the final wash, the cells were resuspended in 100 μL of staining buffer (PBS supplemented with 2% foetal bovine serum) containing fluorochrome-conjugated antibodies: 0.5 μg of anti-F4/80 (clone 123109, BioLegend, San Diego, CA, USA) and 0.25 μg of anti-CD11b (clone 101211, BioLegend, USA). The samples were incubated on ice for 20 min in the dark to allow proper binding of the antibodies to their respective surface antigens. Subsequently, the cells were washed by centrifugation (350× *g*, 5 min), the supernatant was discarded, and 500 μL of fresh staining buffer was added to each tube. The cells were gently and thoroughly resuspended to ensure a single-cell suspension. Flow cytometric analysis was performed using a NovoCyte flow cytometer (Eisenbio, Eisenbio, Sacramento, CA, USA), and data were acquired and analysed using the associated software.

#### 2.4.2. Cell Treatments and Quantitative Real-Time PCR (RT-qPCR)

In order to evaluate the effects of NF on the expression of the main pro-inflammatory markers, IL-6, IL-4, TNF-α, IL-1β, Arg-1, and CD206, quantitative Real-Time PCR (RT-qPCR) was performed after the treatment experiments. Specifically, RAW264.7 cells (1 × 10^4^ cells/well) were treated with NF (40 mg/mL) or placebo for 30 min before being stimulated with 500 ng/mL LPS (Sigma-Aldrich, St. Louis, MO, USA; CAS: 93572-42-0) and 20 ng/mL IFN-γ (Z02916, Ginrys, Nanjing, China) for 6 h. The control was performed by treatment with an equal volume of the cellular medium. After this incubation period, the total RNA of macrophage cells of both M1 and M0 types are extracted using TRI-Reagent (Sigma-Aldrich, Milan, Italy), according to the manufacturer’s instructions, followed by reverse-transcription using iScriptTM Reverse Transcription Supermix (Bio-Rad, Milan, Italy). RT-qPCR was performed using a CFX384 real-time qPCR detection system (Bio-Rad, Milan, Italy). Relative gene expression was obtained by normalising the Ct values of each experimental group against the β-actin transcript level as the housekeeping gene for normalisation using the 2-DCt formula. mRNA levels were expressed as arbitrary units (A.U.). Primer sequences were designed according to the previously reported protocol [30].

IL-6: 5′-CGGAGAGGAGACTTCACAGAG-3′; 5′-ATTTCCACGATTTCCCAGAG-3′
IL-1β: 5′-TACCAGTTGGGGAACTCTGC-3′; 5′ -GGGCCTCAAAGGAAAGAATC-3′
TNF-α: 5′-CAGTAGACAGAAGAGCGTGGT-3′; 5′ -AGGCACTCCCCCAAAAGA-3′
Arg-1: 5′-CTGGTTGTCAGGGGAGTGTT-3′; 5′- GTGAAGAACCCACGGTCTGT-3′
IL-10: 5′-CGGAAACAACTCCTTGGAAA-3′; 5′-AAGTGTGGCCAGCCTTAGAA-3′
Cd206: 5′-AGGACATGCCAGGGTCACCTTT-3′;5′-GTTCACCTGGAATGGTTCTC-3′

### 2.5. Animal Study

#### 2.5.1. Carrageenan-Induced Paw Oedema Model

To evaluate NF anti-oedematous effect, a carrageenan-induced hind paw oedema model was employed in thirty Sprague-Dawley rats (Charles River, Calco, Italy), approximately 4 weeks old. The animals were housed under standard laboratory conditions with a constant temperature (24 ± 1 °C), relative humidity (60 ± 5%), and a 12 h light/dark cycle [31]. The animals were monitored twice daily, and their health status was estimated by a general assessment of animal activity, external appearance, and absence of disease. Food and water intake were provided ad libitum. The animals were allowed to acclimatise for at least one week before randomisation and treatment. All the procedures were performed in accordance with the NIH Guide for the Care and Use of Laboratory Animals (NIH Publication No. 68-23, revised 1985) and approved by the local Institutional Animal Care and Use Committee (IACUC), Ministero della Salute, Direzione Generale della Sanità Animale e dei Farmaci Veterinari Ufficio 6 (protocol n. 354/2019-PR; approval date: 14 May 2019). The study adhered to the ARRIVE guidelines and the National Research Council’s Guide for the Care and Use of Laboratory Animals. The animals were randomly assigned to experimental groups (*n* = 10 per group; *n* = 30 for the three groups of the total experimental design) using a random number generator (Excel RAND function). An a priori power analysis was conducted for both models with three parallel groups (1:1:1), considering an α = 0.05 and 80% power. No a priori inclusion or exclusion criteria were defined, and no animals or data points were excluded from the analysis. All the outcome assessments were performed in a blinded manner, with treatment administration carried out by an operator not involved in data collection or analysis. The randomisation sequence was computer-generated by an independent operator using variable block sizes (3 and 6), with allocation concealment ensured by sequentially numbered, opaque, sealed envelopes containing anonymised treatment codes (A/B/C). The study materials (treatment and vehicle) were pre-labelled and indistinguishable in appearance/handling. The dosing staff only knew the codes; all the outcomes were evaluated in the study, and statisticians remained blinded until the database was locked. The primary outcome of the overall experimental design was the reduction in oedema, assessed as paw thickness in the carrageenan-induced paw oedema model.

Oedema was induced by subplantar injection of 0.1 mL of 1% (*w*/*v*) carrageenan solution (in sterile saline) into the right hind paw of each rat. The animals were randomly divided into three groups: placebo (*n* = 10), carrageenan control (*n* = 10; CAS: 9000-07-1), and NF-treated group (*n* = 10). The placebo group received an oral administration of vehicle (0.5% sodium carboxymethyl cellulose in water; CAS: 9004-32-4), while the NF-treated group received a single oral dose of the nutraceutical formulation (40 mg/mL, 1 mL) one hour before carrageenan injection. The control group received carrageenan without any pretreatment. Paw thickness was measured at 0, 1, 3, and 6 h post-injection using a digital calliper. Measurements were taken at the same anatomical location on the injected paw to assess the development and resolution of oedema over time. The change in paw thickness was used as an index of the inflammatory response and the efficacy of the treatment. All the procedures were designed to minimise animal pain, suffering, and distress. The animals were closely monitored throughout the experimental period, and no signs of severe or persistent discomfort or adverse events were observed. No analgesic treatment was administered in order to avoid interference with inflammatory endpoints, in accordance with ethical approval. Given the acute and short-term nature of the experimental models, no specific humane endpoints beyond continuous clinical monitoring were predefined.

#### 2.5.2. Assessment of Anti-Inflammatory Activity in an Acetic Acid-Induced Inflammation Model

Thirty Sprague-Dawley rats (Charles River, Calco, Italy), approximately 4 weeks old, were used to evaluate the anti-oedematous effects of a nutraceutical formulation (NF) in a model of acetic acid (AA)-induced peritoneal inflammation. All the animals were housed under controlled environmental conditions, including a constant temperature (24 ± 1 °C), relative humidity (60 ± 5%), and a 12 h light/dark cycle. The animals were monitored twice daily, and their health status was estimated by a general assessment of animal activity, external appearance, and absence of disease. Food and water intake were provided ad libitum. The animals were allowed to acclimatise for at least one week before randomisation and treatment. All the procedures were performed in accordance with the NIH Guide for the Care and Use of Laboratory Animals (NIH Publication No. 68-23, revised 1985) and approved by the local Institutional Animal Care and Use Committee (IACUC) (protocol n. 354/2019-PR). The study adhered to the ARRIVE guidelines and the National Research Council’s Guide for the Care and Use of Laboratory Animals. The animals were randomly assigned to experimental groups (*n* = 10 per group; *n* = 30 for the three groups of the total experimental design) using a random number generator (Excel RAND function). An a priori power analysis was conducted for both models with three parallel groups (1:1:1), considering an α = 0.05 and 80% power. No a priori inclusion or exclusion criteria were defined, and no animals or data points were excluded from the analysis. All the outcome assessments were performed in a blinded manner, with treatment administration carried out by an operator not involved in data collection or analysis. The randomisation sequence was computer-generated by an independent operator using variable block sizes (3 and 6), with allocation concealment ensured by sequentially numbered, opaque, sealed envelopes containing anonymised treatment codes (A/B/C). The study materials (treatment and vehicle) were pre-labelled and indistinguishable in appearance/handling. The dosing staff only knew the codes; all the outcomes were evaluated in the study, and statisticians remained blinded until the database was locked. The placebo group received an oral administration of distilled water containing 0.5% sodium carboxymethyl cellulose. The AA group (inflammation control) received an intraperitoneal injection of 400 μL of 0.5% (*v*/*v*) acetic acid (CAS: 64-19-7) to induce an inflammatory response. The NF treatment group received a single oral dose of the nutraceutical formulation (40 mg/mL, 1 mL) one hour before the induction of inflammation with AA. All the procedures were designed to minimise animal pain, suffering, and distress. The animals were closely monitored throughout the experimental period, and no signs of severe or persistent discomfort or adverse events were observed. No analgesic treatment was administered in order to avoid interference with inflammatory endpoints, in accordance with ethical approval. Given the acute and short-term nature of the experimental models, no specific humane endpoints beyond continuous clinical monitoring were predefined. The secondary outcomes were evaluated to investigate mechanistic aspects related to inflammation and vascular permeability, including plasma leukotriene B4 (LTB4) levels and total plasma protein concentration. Twenty minutes after AA injection, blood samples were collected from the caudal vein and centrifuged at 400× *g* for 8 min at 4 °C to obtain plasma. The supernatant was used for the quantification of LTB4 and total protein levels.

Total protein concentration in plasma was measured using a colorimetric assay (Bio-Rad DC Protein Assay, Bio-Rad, Hercules, CA, USA; code 5000111) based on the reaction of proteins with an alkaline copper tartrate solution and Folin reagent. In brief, 50 μL of plasma was added to each well of a 96-well microplate (NUNC™ Brand Products, Roskilde, Denmark), followed by 25 μL of alkaline copper tartrate solution and 200 μL of diluted Folin reagent. After incubation for 15 min at room temperature, absorbance was read at 700 nm using a microplate reader. A bovine serum albumin (BSA; CAS: 9048-46-8) standard curve was used for quantification. LTB4 levels were determined using a competitive ELISA kit (Cayman Chemical, Ann Arbor, MI, USA; code: 520111) by adding 50 μL of plasma to each well of a 96-well ELISA plate and following the assay protocol as per the manufacturer’s instructions.

### 2.6. Statistical Analysis

All data are expressed as mean ± standard deviation (SD) from at least three independent experiments. Statistical comparisons between groups were performed using one-way ANOVA followed by Tukey’s multiple comparisons test. A *p*-value < 0.05 was considered statistically significant. In addition to *p*-values, Cohen’s *d* was used to quantify effect size, where applicable.

## 3. Results

### 3.1. Antioxidant and Anti-Inflammatory Activity of NF

**Figure 1:**
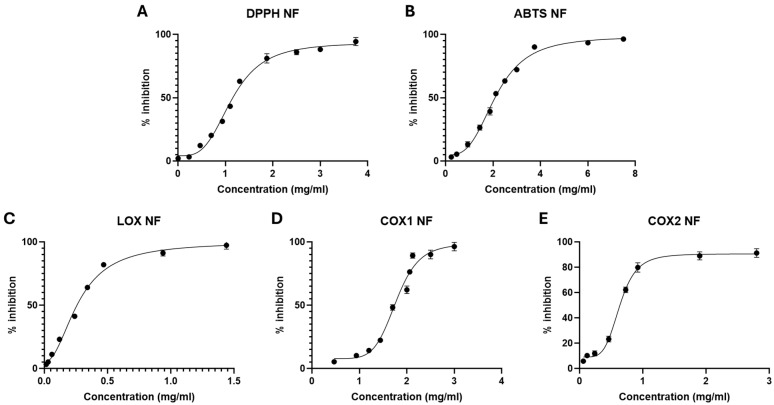
(**A**) Antioxidant activity of NF expressed as IC_50_ by DPPH assay. (**B**) Antioxidant activity of NF expressed as IC_50_ by ABTS assay. (**C**) Anti-inflammatory activity of NF evaluated by the inhibition of 5-LOX activity. (**D**) Anti-inflammatory activity of NF evaluated by the inhibition of COX-1 activity. (**E**) Anti-inflammatory activity of NF evaluated by the inhibition of COX-2 activity. Data are expressed as mean ± SD of at least three independent experiments (*n* = 3).

To preliminarily characterise NF biological activity to counteract oxidative stress and inflammation, its antioxidant and enzyme inhibitory activities were evaluated using in vitro assays. As shown in Figure 1A,B, NF exhibited a concentration-dependent radical scavenging activity in both the DPPH and ABTS assays, with IC_50_ values of 1.14 mg/mL (95% CI 1.19–1.40) and 2.13 mg/mL (95% CI 2.06–2.20), respectively. In parallel, the anti-inflammatory potential of NF was assessed by measuring its inhibitory effects on key enzymes involved in the arachidonic acid cascade. As reported in Figure 1C, NF significantly inhibited 5-lipoxygenase (5-LOX) activity in a dose-dependent manner, yielding an IC_50_ value of 0.27 mg/mL (95% CI 0.25–0.29), indicative of a strong interference with leukotriene biosynthesis. Zileuton was used as the reference anti-inflammatory compound, with an IC_50_ value of 0.12 µg/mL (95% CI 0.095–0.145). Furthermore, NF demonstrated inhibitory activity against both cyclooxygenase isoforms, COX-1 and COX-2 (Figure 1D and Figure 1E, respectively). The inhibition curves revealed a clear concentration-dependent effect, with measurable IC_50_ values for both enzymes of 1.78 mg/mL (95% CI 1.71–1.84) and 0.64 mg/mL (95% CI 0.61–0.66), respectively. Naproxen was used as the reference anti-inflammatory compound, with IC_50_ values of 0.004 mg/mL (95% CI 0.0015–0.0065) and 0.003 mg/mL (95% CI 0.0005–0.0055) for COX-1 and COX-2, respectively.

### 3.2. Inhibitory Effects of NF on the Macrophage M1 Polarisation

**Figure 2:**
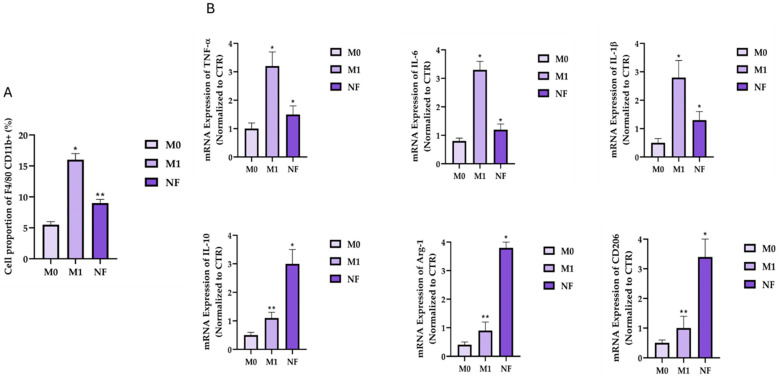
(**A**) Effect of NF treatment on the proportion of F4/80^+^CD11b^+^ cells among M1-type macrophages by flow cytometry. (**B**) Effect of NF treatment on the mRNA expression levels of pro- and anti-inflammatory markers (IL-6, TNF-α, IL-1β, IL-10, Arg-1, and CD206) in RAW264.7 macrophages polarised to the M1 phenotype. The cells were treated with NF (40 μg/mL) for 6 h. Data are expressed as mean ± SD of at least three independent experiments (*n* = 3). Statistical analysis was performed using one-way ANOVA followed by Tukey’s multiple comparisons test (* *p* < 0.05, ** *p* < 0.01 vs. M1 group).

First, to evaluate the nutraceutical formulation’s ability to reduce the oedema-related inflammatory process, its capacity to decrease the proportion of F4/80^+^CD11b^+^ cells among M1-type macrophages was investigated. Specifically, our results indicated that the overall proportion of F4/80^+^CD11b^+^ cells among pro-inflammatory M1 macrophages increased significantly (*p* < 0.01), 3.23 times higher than that of the M0 cells group (Figure 2A). Generally, the markers F4/80 and CD11b are commonly associated with macrophages in their M1 state. F4/80 is a well-established marker of mouse macrophages, and CD11b is involved in the migration and adhesion of these cells, facilitating their accumulation at inflammatory sites. By measuring the reduction in cells expressing both F4/80 and CD11b, researchers can specifically assess the impact of a nutraceutical formulation on the presence and activity of pro-inflammatory M1-type macrophages. The rationale behind studying this parameter lies in the potential of the nutraceutical formulation to shift the balance away from the M1 pro-inflammatory state towards a more anti-inflammatory or regulatory state, possibly the M2 type [32]. Interestingly, the proportion of F4/80^+^CD11b^+^ M1 cells in the treatment group was lower than that in the positive control group (43.7%, 95% CI 34.9–52.5, *p* < 0.05), indicating that NF positively affected the polarisation of RAW264.7. Additionally, to corroborate the NF activity in inducing the polarisation of M1-type macrophages into M2-type, the expression level of the main inflammatory and anti-inflammatory markers was investigated and quantified by RT-qPCR analysis. Specifically, the quantitative analysis of such markers was investigated in both groups M0 and M1. Our results indicate that the expression of pro-inflammatory mediators, i.e., TNF-α, IL-6, and IL-1b were drastically reduced after NF treatment, with a calculated significant reduction (*p* < 0.001 vs. M1 group) of 51.3% (95% CI 48.0–58.7), 64.1% (95% CI 57.0–71.2), and 53.7% (95% CI 51.7–55.7), respectively. In the same manner, regarding the mRNA expression level of the main investigated anti-inflammatory mediators, a significant increase was observed, with the highest increase calculated for Arg-1 (Figure 2B). These results indirectly suggest a potential polarisation of macrophage M1-type into M2-type.

### 3.3. Effect of NF on Plasmatic LTB4 and Protein Levels in Acetic Acid-Induced Inflammation

**Figure 3:**
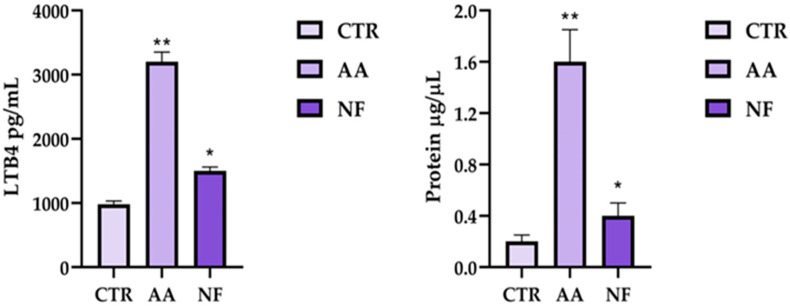
LTB4 and protein plasma levels in rats treated with NF. Data are expressed as mean ± SD of at least independent experiments (*n* = 10 rats per group). Statistical analysis was performed using one-way ANOVA followed by Tukey’s multiple comparisons test (* *p* < 0.05 and ** *p* < 0.01 vs. AA). Effect sizes were calculated using Cohen’s *d*, confirming large effects (LTB4: *d* = 1.45; plasma proteins: *d* = 1.73).

To confirm the modulating effect of NF on the production of key oedema-related inflammatory markers, its ability to control acetic acid injection-induced inflammation in rat models was investigated by examining LTB4 and plasma protein levels in the treated rats. Specifically, following treatment, a significant reduction in plasma levels of LTB4 (*p* < 0.01, Cohen’s *d* = 1.45) and plasma proteins (*p* < 0.01, Cohen’s *d* = 1.73) was observed compared to the control group (*n* = 10) treated with carboxymethylcellulose (*n* = 10) and the AA group (*n* = 10), suggesting that NF effectively modulates the inflammatory response. In detail, plasma LTB4 levels increased from 930 ± 80 pg/mL in the control group to 3150 ± 250 pg/mL in the AA-treated animals, while NF pretreatment significantly reduced LTB4 concentrations to 1450 ± 100 pg/mL (Figure 3). Similarly, total plasma protein levels rose from 0.25 ± 0.05 mg/mL in the control rats to 1.60 ± 0.30 mg/mL following AA administration, whereas NF treatment markedly attenuated this increase, reducing protein levels to 0.40 ± 0.15 mg/mL (Figure 3). Additionally, the decrease in LTB4 levels suggests a potential mechanistic contribution through which NF may be associated with reduced vascular permeability and oedema formation, leading to a decrease in plasma protein concentration in the treatment group. These findings support the hypothesis that NF possesses valuable anti-inflammatory properties, providing a promising avenue for further exploration in managing inflammation-related conditions.

### 3.4. NF Reduces Paw Oedema in a Carrageenan-Induced Rat Model

**Figure 4:**
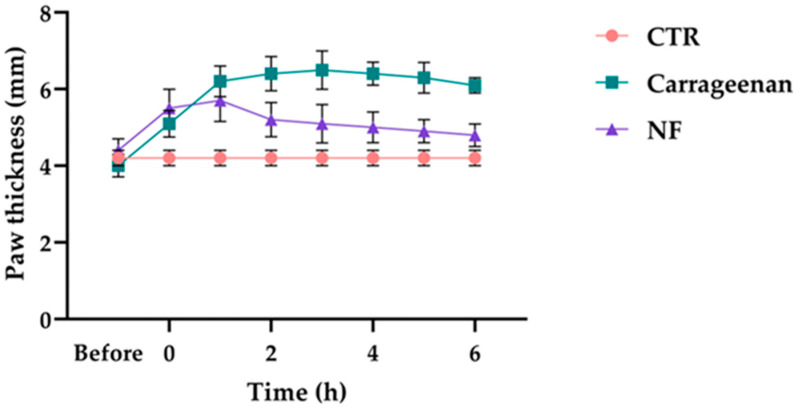
Effect of NF treatment on paw thickness (mm) in the carrageenan-induced rat paw oedema model. The rats were pretreated with NF (40 μg/mL, 1 mL, orally) 1 h before carrageenan injection. Paw thickness was measured at 0, 1, 3, and 6 h post-injection. Data are expressed as mean ± SD of at least independent experiments (*n* = 10 rats per group).

The anti-oedematous potential of NF was investigated in a carrageenan-induced rat oedema model. Specifically, injection of carrageenan into the hind paws of rats resulted in oedema as measured by the thickness of the paws. As our results show, the paw thickness in the placebo group (*n* = 10) was 4.32 ± 0.10 mm (95% CI 4.07–4.56) after 0 h and remained constant until the end of 6 h. In the carrageenan control group (*n* = 10), the initial paw thickness was 5.27 ± 0.10 mm (95% CI 5.01–5.53), reached its maximum of 7.07 ± 0.42 mm (95% CI 6.02–8.12) after 3 h, and decreased to 5.64 ± 0.71 mm (95% CI 3.87–7.40) after 6 h. In the treatment group (*n* = 10), the animals had an initial paw thickness of 5.36 ± 0.40 mm (95% CI 4.36–6.36), which increased to 5.97 ± 0.27 mm (95% CI 5.30–6.64) after 1 h and gradually decreased to 5.01 ± 0.53 mm (95% CI 3.69–6.33), 4.69 ± 0.38 mm (95% CI 3.74–5.64), and 4.25 ± 0.21 mm (95% CI 3.71–4.78) after 2, 4, and 6 h, respectively (Figure 4). Overall, our results indicate that the NF treatment induces a progressive reduction in paw thickness compared to a carrageenan group, with a general reduction in oedema volume of −22.5% vs. control (95% CI 11.0–34, *p* < 0.01, Cohen’s *d* = 2.65).

## 4. Discussion

Oedema is a non-resolving swelling pathological state caused by a sustained imbalance between microvascular filtration, endothelial barrier integrity, lymphatic clearance, and an inflammatory condition [33]. Patients living with chronic oedema experience a substantial disease burden, with documented impairment in health-related quality of life (QOL) [34]. Principally, a key factor that influences the various described oedema phenotypes is dysfunction in the vascular and inflammatory barriers. Increased permeability of the endothelium promotes the leakage of fluid and plasma proteins, which sustains the recruitment of leukocytes and chemokines and inflammation. Once established, oedema can function as a pathophysiological amplifier by altering interstitial pressure gradients and interfering with the extracellular matrix (ECM)-dependent regulation of fluid transport, leading to persistent tissue damage. This condition, in turn, sustains the recruitment and activation of macrophage populations responsible for the clearance of cellular and matrix-derived detritus. When these macrophage-mediated reparative programmes become dysregulated, they undergo phenotypic polarisation toward pro-fibrogenic and pro-adipogenic states, characterised by enhanced expression of TGF-β–driven transcriptional circuits, inflammatory mediators, secretion of extracellular matrix components, and promotion of preadipocyte differentiation. This pathological remodelling process is particularly evident in lymphoedema, a prototypical chronic oedema phenotype marked by sustained lymph stasis, persistent low-grade inflammation, progressive fibrosis, and adipose tissue expansion [35,36,37].

Given the multifactorial nature of this pathological condition, which involves functional alterations of the lymphatic system, the expression of inflammatory mediators, immune cell tissue infiltration, and protein accumulation in the interstitial fluid [38], the main goal of the current project is to formulate an innovative nutraceutical preparation capable of modulating the various pathways involved in the onset of such complex pathology. This study was designed to provide an integrated, mechanism-informed evaluation of a multicomponent nutraceutical formulation across complementary experimental models relevant to inflammation-driven oedema. In this context, an additional and highly relevant aspect of oedema pathophysiology is represented by the tight interplay between oxidative stress and inflammatory signalling. The excessive generation of reactive oxygen species is known to exacerbate endothelial dysfunction, promote lipid mediator synthesis, and amplify vascular permeability, thereby sustaining tissue swelling and inflammatory cell recruitment [39]. Accordingly, the ability to modulate both redox balance and inflammatory mediator production represents a key therapeutic objective in the management of oedema-related disorders.

Although several nutraceutical ingredients and combinations containing polyphenols, coumarins, tannins, and bromelain have been previously investigated in the context of inflammation and oedema, the present study was designed to address the multifactorial nature of oedema through an integrated experimental approach. Rather than focusing on a single biological endpoint, the nutraceutical formulation was conceived through a pathway-oriented selection of bioactive constituents, in which individual components preferentially target distinct pathological processes, and was evaluated across complementary biochemical, cellular, and in vivo models. This approach allowed the assessment of oxidative stress modulation, inhibition of arachidonic acid-derived mediators, macrophage polarisation, vascular permeability, and oedema formation. The consistency of these effects across different levels of pathophysiology supports a coordinated, multi-target biological activity of the formulation, providing mechanistic insight beyond the symptomatic evidence reported for individual components or previously described mixtures. Therefore, the present formulation was designed to exert a dual antioxidant and anti-inflammatory activity, as supported by its capacity to scavenge free radicals and to inhibit key enzymes involved in the arachidonic acid cascade. The concurrent modulation of lipoxygenase- and cyclooxygenase-dependent pathways is particularly relevant, as leukotrienes and prostaglandins act in concert to promote leukocyte chemotaxis, endothelial barrier disruption, and persistence of inflammation. Targeting these enzymatic systems may thus contribute to limiting the biochemical amplification of the inflammatory response that characterises oedematous tissues [40].

From a compositional standpoint, these effects may arise from the complementary biological activities of the bioactive constituents of NF. Moreover, the present study was not designed to deconvolute the contribution of individual components or to demonstrate pharmacological synergy among them. Rather, NF was investigated as a single functional entity with the aim of evaluating its multi-target biological activity across complementary pathogenic pathways relevant to oedema. Polyphenol-rich extracts from *Olea europaea* L. and *Quercus robur* L. are widely reported to exert strong antioxidant activity and to interfere with pro-inflammatory enzyme systems through both redox-dependent and direct inhibitory mechanisms. Hydroxytyrosol has been shown to modulate lipid mediator biosynthesis and to counteract oxidative damage at the vascular level, while tannins are known to regulate inflammatory signalling and enzyme activity [41]. Coumarin-containing extracts from *Melilotus officinalis* L. contribute further anti-inflammatory and vasoprotective properties [21], whereas bromelain has been extensively described for its capacity to attenuate inflammatory cascades and vascular permeability [42].

Overall, the antioxidant and enzyme inhibitory profile of NF provides a mechanistic basis for its ability to counteract the early biochemical events that precede immune cell activation and tissue infiltration. By limiting oxidative stress and lipid mediator-driven inflammation, NF could produce a microenvironment less permissive to sustained inflammatory activation, thereby setting the stage for downstream modulation of immune cell behaviour.

In addition, oedema is accompanied by the upregulation of inflammatory factors, which, in turn, induce the infiltration of activated M1 macrophages able to clear tissue fragments from the damaged site. Physiologically, M1-type macrophages serve as the primary line of defence against external threats [43], but simultaneously they produce a large amount of pro-inflammatory mediators, including IL-6, IL-1β, and TNF-α [44]. The continuous release of such mediators at the stasis level induces a further infiltration of M1 macrophages that leads to severe tissue injury and fibrosis [32,45]. To explore the impact of NF-based treatment on macrophage polarisation, we investigated its effect on the proportion of F4/80^+^CD11b^+^ cells, representing the mature subpopulation of M1-type macrophages. Our findings suggest that NF treatment significantly reduced the F4/80^+^CD11b^+^ cell population within M1 macrophages, suggesting a decrease in M1 polarisation from M0 macrophages, which are the native, non-inflammatory type. Our evidence is perfectly in agreement with Suwen et al., who have studied the effect of procyanidin C1 and procyanidin fraction from *Castanea mollissima* on the F4/80^+^CD11b^+^ cell population among M1 macrophages [29]. Structurally, procyanidins are polymers of flavan-3-ols, which serve as the fundamental units of tannin polymeric structures [28]. Consequently, the NF tannin fraction, the primary bioactive compound of the *Quercus robur* L. monocomponent, may reasonably exert a significant influence on M1 polarisation similarly [29]. In support of this, other studies have shown that tannin-rich extracts from *Stryphnodendron adstringens* bark significantly downregulate the mRNA expression of F4/80^+^CD11b^+^ macrophage markers in rat models of inflammation, indicating a shift toward the M2 anti-inflammatory phenotype [46]. Moreover, to further confirm the NF ability to convert the inflammation-related oedema, we have investigated the mRNA expression level of the main pro/anti-inflammatory mediators in M1-treated cells. Interestingly, our results highlight that NF treatment was simultaneously able to reduce the production of pro-inflammatory cytokines, such as TNF-α, IL-1b, and IL-6, and to increase the production of anti-inflammatory cellular markers, such as IL-10, CD206, and Arg-1. These results indirectly corroborate the hypothesis that NF treatment may modulate macrophage polarisation. Generally, Arg-1, CD20, and IL-10 are inflammatory markers characteristic of the M2 macrophage type, while the production of pro-inflammatory mediators, IL-6, IL-1β, and TNF-α, was positively correlated with the shift polarisation to M1-type. This expression profile supports the hypothesis that NF may modulate macrophage polarisation from the M1 to the M2 phenotype. Notably, IL-10, CD206, and Arg-1 are canonical markers of M2 macrophages, while IL-6, IL-1β, and TNF-α are positively associated with M1 polarisation. Given the multicomponent composition of NF, several active constituents could be responsible for these observed effects. For example, rubiadin, the primary bioactive compound of the *Morinda citrifolia* L. leaf extract, has been shown to reduce transcriptional levels of pro-inflammatory cytokines [47]. Similarly, coumarin-rich extracts from *Melilotus officinalis* L. have demonstrated comparable anti-inflammatory properties [21]. Additionally, our results are perfectly in line with other findings, which reported that hydroxytyrosol-based treatments can inhibit the LPS-induced overexpression of F4/80 and mRNA expression of M1 macrophage markers TNF-α, IL-6, and IL-1β, and simultaneously upregulate the mRNA levels of M2 macrophage markers, such as IL-10 and Arg1 [48]. While these transcriptional changes provide valuable insight into the inflammatory polarisation state, it should be noted that mRNA expression does not necessarily reflect protein abundance or activity. Therefore, protein-level analyses would be required to further corroborate these observations.

Having established the role of NF in modulating macrophage polarisation and cytokine production, we next investigated its systemic anti-inflammatory effects in vivo using an acetic acid-induced inflammation model in rats. Pretreatment with NF (1 h before AA injection) significantly reduced both total plasma protein levels and LTB4 concentration. Our results highlight that the NF pre-treatment of 1h before the induction of the inflammatory process by acetic acid injection in a rat model was able to reduce the total proteins and plasmatic LTB4 levels. In inflammatory oedema, elevated leukotriene B4 (LTB4) can amplify leukocyte activation and promote microvascular barrier dysfunction, thereby favouring plasma protein leakage and tissue swelling [49,50]. Accordingly, several pieces of evidence indicate that LTB4 is a mediator deeply implicated in oedema formation in settings where vascular permeability is increased. The NF-based pretreatment reduced the LTB4 production by 53% vs. the AA group, highlighting the multi-target nature of the formulated product. This result was corroborated by the in vitro assay of lipoxygenase inhibition by NF, which indicated a strong direct inhibitory activity of the enzyme with an experimentally calculated IC_50_ of 0.27 mg/mL. Our results align with other findings, where the activity of natural compounds, such as hydroxytyrosol, inhibits the LTB4-mediated lymph accumulation effects. A recent review described that this compound can directly inhibit the 5-Lipoxygenase enzyme (5-LO), a key molecular player involved in the inflammatory-related lymphoedema process. Specifically, it was well reported that hydroxytyrosol is able to directly contrast the 5-LO activity by binding to iron ions and reducing them to the catalytically inactive ferrous form. The inhibition of LTB4 synthesis by hydroxytyrosol is crucial in treating chronic lymphatic oedema phenotypes. This evidence is further corroborated by additional in vivo results, reporting that Ketoprofen, Zileuton, and Bestatin treatment decreases the LTB4 production and ameliorates lymphoedema conditions [51], supporting the translational relevance of modulating this pathway in chronic, inflammation-sustained oedema states. We also evaluated NF’s ability to restore vascular function by measuring plasma protein levels, a commonly accepted indicator of capillary integrity. NF treatment significantly reduced capillary permeability, as indicated by a 48.8% decrease in plasma protein levels compared to the AA-induced group. Among the active components of NF, bromelain may contribute to this effect. This proteolytic enzyme complex derived from *Ananas comosus* L. is well known for its anti-oedematous and anti-inflammatory properties [52]. Previous studies have shown that bromelain reduces vascular permeability and oedema in various inflammatory models, including bilateral common carotid artery occlusion in rats [53]. These effects are thought to be mediated, at least in part, by the reduction in systemic inflammation. When inflammation occurs, vascular permeability increases, allowing proteins and fluids to leak into tissues and contribute to swelling and pain. NF, by attenuating the inflammatory response, may reduce this protein extravasation, thereby alleviating oedema. Finally, the anti-oedema activity of the prepared formulation was assayed in the carrageenan-induced rat paw oedema model. Our results highlight that the NF treatment reduces the Rat Paw Thickness (mm), with a calculated percentage of paw oedema inhibition by 22% in comparison to the carrageen group. These data are perfectly in line with the findings of other authors, who have described that a *Melilotus officinalis* L. extract-based topical formulation was able to reduce the inflammation-related in carrageenan-induced rat paw oedema model [54]. Finally, these data support the potential of NF for treating the various physiopathological changes typically associated with oedema, including modulation of inflammatory mediators and improvement in vascular permeability. This study allowed us to investigate and validate the effect of the formulation on these key processes individually, providing proof of concept for its multicenter potential. From a translational perspective, the use of a standardised multicomponent nutraceutical formulation allows reproducible evaluation of biological activity within a nutritional framework. Rather than aiming at pharmacological substitution, the present findings support the rationale for complementary, multi-target supplement strategies addressing inflammation-driven vascular alterations. This approach is particularly relevant in the field of nutraceutical research, where complex pathophysiological conditions are often modulated through integrated dietary-derived interventions. However, some limitations of the present study should be acknowledged. First, the in vivo experiments were performed using well-established acute models of inflammation and oedema, which are appropriate for investigating early inflammatory events and vascular permeability changes but may not fully capture the complexity and chronic progression of oedema-related conditions in humans. In particular, long-term processes such as tissue remodelling, fibrosis, and lymphatic dysfunction cannot be completely reproduced within short-term experimental settings. Accordingly, while the present findings provide mechanistic and functional evidence supporting the ability of NF to modulate key inflammatory and vascular pathways involved in oedema formation, they should not be interpreted as direct evidence of efficacy in chronic oedema. Rather, these results offer a biological and pathophysiological rationale that may inform future investigations using chronic disease models or clinical studies specifically designed to address long-standing oedematous conditions. Second, although rigorous randomisation, blinding procedures, and predefined experimental protocols were applied to ensure robustness and reproducibility, biological variability inherent to in vivo models may contribute to variability in certain outcome measures. In addition, the present study was designed to provide an integrated evaluation of the anti-inflammatory and anti-oedematous effects of NF across biochemical, cellular, and in vivo models. Although the results consistently indicate modulation of macrophage polarisation and reduction in arachidonic acid-derived inflammatory mediators, the mechanistic interpretation of these findings is based on associative evidence. The study did not include direct validation of specific signalling pathways, receptor-level interactions, or loss-of-function approaches. Finally, while the present findings provide consistent mechanistic and functional evidence supporting the multi-target activity of the nutraceutical formulation, confirmation of these effects in human subjects will require well-designed clinical trials. In this context, it should be noted that the experimental doses and concentrations employed in the present in vitro and in vivo models were not supported by pharmacokinetic data and were not designed to reflect achievable human exposure levels. Accordingly, clinical studies will be essential not only to confirm efficacy but also to establish appropriate dose translation, bioavailability, optimal dosing regimens, and long-term safety in the context of oedema-related disorders.

## 5. Conclusions

In conclusion, this study highlights the therapeutic potential of the novel nutraceutical formulation EDEMIX^®^ in the management of oedema, with particular emphasis on its anti-inflammatory properties. Our findings demonstrate that NF effectively modulates macrophage polarisation by reducing the proportion of F4/80^+^CD11b^+^ cells within the M1 macrophage population and simultaneously rebalancing the expression of key inflammatory mediators, downregulating M1-associated cytokines (e.g., TNF-α, IL-1β, and IL-6) and upregulating M2 markers (e.g., IL-10, Arg-1, and CD206). Moreover, NF significantly decreased plasma levels of LTB4, a crucial mediator implicated in the onset and progression of lymphoedema, and improved vascular function, as evidenced by the reduction in plasma protein leakage in an acetic acid-induced inflammation model. The formulation also demonstrated potent anti-oedematous activity in vivo, as shown by the significant reduction in paw swelling in the carrageenan-induced oedema model. Taken together, these results support the potential of NF for the management of the distinct physiopathological alterations typically involved in such pathological states, including modulation of inflammatory mediators, reduction in oedema, and improvement in vascular permeability. This study allowed us to explore and validate the formulation’s activity on these key processes individually, offering proof of concept of its multi-targeted potential.

## Data Availability

The original contributions presented in the study are included in the article, further inquiries can be directed to the corresponding author.
